# 超高效液相色谱-静电场轨道离子阱高分辨质谱法测定设施菜地土壤中有机磷酸二酯类化合物

**DOI:** 10.3724/SP.J.1123.2022.05002

**Published:** 2023-01-08

**Authors:** Mengfei LIU, Mei WANG, Ang ZHAO, Lin ZHU, Chun WANG, Chao WEI, Wei LIU, Jianzhong XU

**Affiliations:** 1.河北大学化学与环境科学学院, 河北 保定 071002; 1. College of Chemistry & Environment Science of Hebei University, Baoding 071002, China; 2.阻燃材料及加工技术工程技术研究中心, 河北 保定 071002; 2. The Flame Retardant Material and Processing Technology Engineering Research Center of Hebei Province, Baoding 071002, China

**Keywords:** 超高效液相色谱-静电场轨道离子阱高分辨质谱, 有机磷酸二酯, 设施菜地土壤, ultra-high performance liquid chromatography-electrostatic field orbitrap high resolution mass spectrometry (UHPLC-Orbitrap HRMS), organophosphate diesters (Di-OPEs), facility vegetable soils

## Abstract

有机磷酸二酯(Di-OPEs)被认为是有机磷酸三酯的生物或非生物降解产物。该研究基于超高效液相色谱-静电场轨道离子阱高分辨质谱(UHPLC-Orbitrap HRMS)建立了设施菜地土壤中磷酸二(2-氯)乙酯(BCEP)、磷酸二(1,3-二氯异丙基)酯(BDCP)、磷酸二正丁酯(DnBP)、磷酸二苯酯(DPhP)和磷酸二(2-乙基己基)酯(DEHP)5种Di-OPEs的定性定量分析方法。首先,土壤样品以甲醇为提取溶剂,超声提取,选择Oasis WAX固相萃取柱进行净化,用8 mL含5% (v/v)氨水的甲醇溶液洗脱,洗脱液浓缩定容后,应用Thermo Accucore RP-MS色谱柱,以甲醇-0.2 mmol/L乙酸铵水溶液为流动相进行分离,采用UHPLC-Orbitrap HRMS测定,质谱分析采用电喷雾负离子模式电离,在全扫描模式下检测。在优化的分析检测条件下,5种Di-OPEs的检出限为0.001~0.047 ng/g,定量限为0.004~0.156 ng/g。5种Di-OPEs的标准曲线线性关系良好,相关系数(*r*)为0.9985~0.9999。在5.0、25.0、50.0 ng/g的添加条件下,3种加标水平的回收率为56.9%~133.0%,相对标准偏差为4.4%~18.9%。应用建立的方法对采集的16个设施菜地土壤样品进行分析,结果显示设施菜地土壤中5种Di-OPEs的含量为2.53~6.94 ng/g,所有样品中Di-OPEs的检出率均高于60%。其中,DnBP是设施菜地土壤中的主要污染物,含量范围为1.37~3.20 ng/g,占Di-OPEs总含量的23.4%~68.8%。该方法操作简便,灵敏度高,重复性良好,适用于设施菜地土壤中Di-OPEs的测定,也为今后开展设施菜地土壤中Di-OPEs的环境行为和人体暴露研究提供可靠的技术支持。

## Abstract (en)

Organophosphate diesters (Di-OPEs) are biotic or abiotic degradation products of organophosphate esters (OPEs). Current analytical methods focus on detecting Di-OPEs in human urine. Human exposure to Di-OPEs in environmental matrices has not been systematically studied. Soil plays an important role in the environmental migration and transformation of organic pollutants. Previous studies found that OPEs are ubiquitous in soil. However, few studies reported OPEs metabolite pollution in soil, especially in facility vegetable soil.

In this study, an ultra-high performance liquid chromatography-electrostatic field orbitrap high resolution mass spectrometry (UPHLC-Orbitrap HRMS) method was developed for the determination of five Di-OPEs (bis(2-chloroethyl) phosphate (BCEP), bis(1,3-dichloro-2-propyl) phosphate (BDCP), di-*n*-butyl phosphate (DnBP), diphenyl phosphate (DPhP), and bis(2-ethylhexyl) phosphate (DEHP)) in the facility vegetable soil. The pretreatment process and chromatographic and mass spectrometric conditions were optimized in the present study. Comparative study of the purification effects of different solid-phase extraction columns showed that Oasis WAX cartridge had best purification efficiency for the five Di-OPEs. The cartridge was first activated using 3 mL methanol, 3 mL methanol containing 5% (v/v) ammonia, and 3 mL 0.1 mol/L sodium acetate-acetic acid buffer solution. Then, the cartridge was rinsed with 3 mL of 30% (v/v) methanol aqueous solution, and finally eluted using 8 mL methanol containing 5% (v/v) ammonia. The effects of mobile phase (with respect to solvent composition and flow rate) and column temperature on the shape and intensity of chromatographic peaks were studied. The optimized UHPLC conditions were as follows: chromatographic column, Thermo Accucore RP-MS; column temperature, 30 ℃; mobile phase, 0.2 mmol/L ammonium acetate aqueous solution and methanol; flow rate, 0.2 mL/min. In the UHPLC-Orbitrap HRMS experiment, the five Di-OPEs were analyzed in full MS mode with negative ionization. Instrumental parameters, such as sheath gas and auxiliary gas, were optimized to determine the MS conditions. The optimized Orbitrap HRMS conditions were as follows: heating electrospray ionization source (HESI), full MS mode with negative ionization; scan range, *m/z* 100-500; ion transfer tube temperature, 320 ℃; automatic gain control of target particle count, 1×10^6^; sheath gas flow rate, 8.58 L/min; auxiliary gas flow rate, 17.40 L/min; spray voltage, 3.2 kV; and S-lens voltage, 50 V. The limits of detection and quantification were 0.001-0.047 ng/g and 0.004-0.156 ng/g, respectively. The correlation coefficients of the calibration curve were 0.9985-0.9999. At three spiked levels, 5.0, 25.0, and 50.0 ng/g, the recoveries of the five Di-OPEs ranged from 56.9% to 133.0% with relative standard deviations of 4.4%-18.9%. The established method was applied to the analysis of the five Di-OPEs in 16 facility vegetable soils. The detection frequencies of the five Di-OPEs exceeded 60% in all soil samples, indicating that the Di-OPEs were ubiquitous in the facility vegetable soil. The contents of the five Di-OPEs in the facility vegetable soil samples ranged from 2.53-6.94 ng/g. DnBP (1.37-3.20 ng/g) and DPhP (0.47-2.44 ng/g) were the predominant congeners in the facility vegetable soil samples, accounting for 23.4%-68.8% and 16.3%-35.9% of the five Di-OPEs, respectively. The developed method is simple, sensitive, and reproducible and can be used effectively for the determination of Di-OPEs in soil. The results of this study will be helpful for understanding the environmental behavior of Di-OPEs and their human exposure in facility vegetable soils.

有机磷酸酯(organophosphate esters, OPEs)是磷酸分子上的氢被取代而形成的磷酸衍生物,是一类重要的人工合成化合物。根据取代基团的不同,OPEs可以分为烷基取代、含卤原子的烷基取代和芳香基取代3种类别。OPEs常常作为阻燃剂和塑化剂被物理添加到各种商品(纺织染料、家具、电子产品、涂料、聚氯乙烯塑料和聚氨酯泡沫)当中,易于通过挥发、磨损、淋出等途径从材料的生产和使用过程中释放进入环境^[1,2]^。随着多氯联苯和多溴联苯醚等卤代阻燃剂的陆续禁用和限用,OPEs作为其替代品开始在世界范围内被广泛使用^[3⇓-5]^。相关研究表明,OPEs具有持久性有机污染物的特性,如持久性、长距离迁移性和生物蓄积性,已经在降尘、空气、水、沉积物和土壤等多种环境介质和生物体中被检出^[6⇓⇓⇓⇓⇓-12]^。

环境介质中的OPEs可通过人体或生物体代谢生成有机磷酸二酯(organophosphate diesters, Di-OPEs)或羟基化产物^[13⇓⇓-16]^。OPEs在生物体内主要的代谢途径是通过脱侧链形成二酯类化合物^[17]^。Di-OPEs被认为是OPEs的生物或非生物降解产物。对于环境介质中Di-OPEs的分析检测方法主要有液相色谱-质谱法、液相色谱-串联质谱法、气相色谱-质谱法和气相色谱-串联质谱法等,前处理方法主要为液液萃取、固相萃取、固相微萃取等方法^[18⇓⇓⇓⇓-23]^。李佩等^[22]^采用固相萃取技术进行富集净化,利用液相色谱-三重四极杆质谱联用技术,建立了人体尿液中Di-OPEs的检测方法,化合物的定量限为0.02~0.50 μg/L,除磷酸二乙酯的加标回收率为17.8%~36.2%,其他化合物的加标回收率为60.5%~104.0%。李栋等^[23]^建立了基于自制混合型小柱的样品净化结合高效液相色谱-串联质谱同时测定Di-OPEs和8-羟基-2'-脱氧鸟苷(8-OHdG)的分析方法,OPEs代谢物和8-OHdG的检出范围分别为6.24~46.07 μg/L和5.90~16.71 μg/L,加标回收率分别为52.36%~114.56%和88.63%~97.72%。

目前大多数研究主要集中于尿液中Di-OPEs的检测^[24,25]^,但是对于人体直接从环境介质中摄入Di-OPEs的研究较少。近期的一些研究发现,Di-OPEs广泛存在于环境中,在多种环境介质中被检出。Li等^[26]^在废水、河水和自来水中检测到Di-OPEs。张博钠等^[21]^应用ENVI-18固相萃取柱净化处理,结合超高效液相色谱-串联质谱法开展了小麦样品中Di-OPEs的筛查分析。Du等^[27]^对住宅灰尘的研究发现,灰尘样本中存在Di-OPEs。He等^[28]^在来自澳大利亚的食品和饮用水样品中检测出了两种Di-OPEs。Li等^[29]^在鱼肉中检测到磷酸二(2-氯乙基)酯(BCEP)和磷酸二(1,3-二氯-2-丙基)酯(BDCIPP)等Di-OPEs。

土壤作为基本的环境基质,对环境中有机污染物的迁移转化起着至关重要的作用。土壤中的Di-OPEs不仅可以通过地表径流、雨水冲刷和淋滤等途径污染地表水和地下水,还可以通过挥发进入大气,造成二次污染,同时也可通过食物链循环、呼吸和皮肤接触进入人体,直接危害人体健康。当前,已有研究表明,土壤环境中存在一定程度的OPEs污染^[30⇓-32]^,但是有关土壤环境中Di-OPEs的研究鲜有报道。Di-OPEs的分析检测对于深入研究OPEs在环境中的迁移转化和生态影响有重要意义。因此,本研究依据我国土壤环境介质中OPEs的污染现状,选取检出率较高、含量较大、较为常见且应用广泛的5种OPEs代谢产物:磷酸二(2-氯)乙酯(bis(2-chloroethyl) phosphate, BCEP)、磷酸二(1,3-二氯异丙基)酯(bis(1,3-dichloro-2-propyl) phosphate, BDCP)、磷酸二正丁酯(di-*n*-butyl phosphate, DnBP)、磷酸二苯酯(diphenyl phosphate, DPhP)和磷酸二(2-乙基己基)酯(bis(2-ethylhexyl) phosphate, DEHP)5种Di-OPEs作为研究对象,建立了5种Di-OPEs的超高效液相色谱-静电场轨道离子阱高分辨质谱(UHPLC-Orbitrap HRMS)分析方法,并将该方法应用于设施菜地土壤的检测。研究结果为设施菜地土壤中Di-OPEs的污染现状提供基础数据,对于深入认知Di-OPEs在环境中的迁移转化行为具有重要意义。

## 1 实验部分

### 1.1 仪器、试剂与材料

Ultimate 3000-Q-Exactive Focus超高效液相色谱-高分辨质谱联用仪(Thermo Fisher公司,美国); SB-800DT超声波清洗仪(宁波新芝生物科技有限公司,中国); N-EVAP氮吹仪(Organomation公司,美国); RV 10旋转蒸发仪(IKA公司,德国)。

5种OPEs代谢产物标准品:BCEP(纯度95%)、BDCP(纯度95%)和DEHP(纯度97%)(TRC公司,加拿大); DnBP(纯度99.9%, Dr. Ehrenstorfer公司,德国); DPhP(纯度99%, Acros公司,美国)。

甲醇、甲酸(色谱纯,Thermo Fisher公司,美国), Oasis WAX固相萃取柱(60 mg, 3 mL)和Oasis HLB固相萃取柱(60 mg, 3 mL)购自沃特世科技有限公司,HP-WAX固相萃取柱(60 mg, 3 mL)和HP-HLB固相萃取柱(60 mg, 3 mL)购自武汉蔚启科技有限公司。醋酸钠(分析纯,纯度99.0%)购自天大化工实验厂,氨水(优级纯,纯度25%~28%)购自科密欧有限公司。

### 1.2 Di-OPEs标准溶液的配制

BCEP、BDCP、DnBP、DPhP和DEHP用甲醇配制成质量浓度为100 μg/mL的单标储备液,用移液枪分别量取10 μL单标储备液,用甲醇稀释至1 mL,配成质量浓度为1 μg/mL的混合标准储备液,于-20 ℃条件下储存备用。使用时,用甲醇配制成0.5、1.0、5.0、10.0、50.0、100.0 ng/mL的标准工作溶液。

### 1.3 样品的采集与前处理

#### 1.3.1 样品采集

本研究中的所有土壤样品均为大型塑料拱棚的设施菜地土壤,采集于河北省沧州市青县,该地区是河北省现代瓜菜示范区,设施蔬菜种植面积超过30万亩。本研究中的样品为采集于该示范区中的16个大型设施蔬菜塑料拱棚中0~10 cm的表层土壤样本,土壤类型为黏质壤土。16个拱棚均为一年两季轮种,2~6月份种植甜瓜,7月份休耕,8~10月份种植黄瓜和豆角。样品采集时,用预先清洁的不锈钢铲采集0~10 cm的表层土壤样本。首先,去除表层覆盖物,然后根据五点采样法从同一个塑料拱棚设施菜地中取5个样品混合成一个复合样品作为该采样点的代表性土样。所有的土壤样品收集后放入棕色玻璃瓶中运回实验室。运输至实验室后,去除土壤样品中的石头和残根,经冷冻干燥后,研磨过150目不锈钢筛,然后用锡箔纸包裹密封保存。所有样品在分析前均保存在-20 ℃冰箱中。

#### 1.3.2 样品提取

准确称取2.0 g设施菜地土壤样品,转移到50 mL玻璃管中,以30 mL甲醇作提取液,超声20 min。提取液经低速离心15 min后将上清液转移至鸡心瓶中,重复提取3次,合并后的提取液经旋转蒸发仪浓缩至约1~2 mL,然后用超纯水稀释到10 mL,用醋酸钠缓冲液(pH 4.5)调节pH至5。

#### 1.3.3 样品净化

样品采用Oasis WAX柱(60 mg, 3 mL)进行萃取分离。固相萃取柱依次用3 mL甲醇、3 mL含5%氨水的甲醇溶液、3 mL 0.1 mol/L醋酸钠-醋酸(NaAc-HAc)缓冲溶剂进行活化,上样后,用3 mL 30%甲醇水溶液淋洗,最后用8 mL含5%氨水的甲醇溶液洗脱目标物。将洗脱液经旋转蒸发仪浓缩至约1~2 mL,然后进行缓慢氮吹,最后交换至500 μL甲醇中待测。

### 1.4 仪器条件

色谱柱为Thermo Accucore RP-MS柱(100 mm×2.1 mm, 2.6 μm),柱温:30 ℃,流动相为0.2 mmol/L乙酸铵水溶液(A)和甲醇(B),流速:0.2 mL/min,进样体积:10 μL。流动相梯度:0~1.0 min, 10%B; 1.0~1.5 min, 10%B~100%B; 1.5~7.0 min, 100%B; 7.0~8.0 min, 100%B~10%B; 8.0~10.0 min, 10%B。

采用可加热电喷雾电离源(HESI),分析物在负离子扫描下以全扫描(Full MS)模式分析,扫描范围:*m/z* 100~500,离子传输管温度:320 ℃,自动增益控制目标粒子数:1×10^6^,鞘气流速:8.58 L/min,辅助气流速:17.40 L/min,喷雾电压:3.2 kV,叠环离子导向器(S-lens)电压:50 V;加热温度:350 ℃。5种Di-OPEs的其他质谱参数见表1。

**表1:** 5种Di-OPEs的质谱参数

Compound	CAS No.	Molecular formula	Retention time/min	Qualitative/quantitative ion
Bis(2-chloroethyl) phosphate (BCEP)	3040-56-0	C_4_H_9_Cl_2_O_4_P	1.10	220.95427
Bis(1,3-dichloro-2-propyl) phosphate (BDCP)	72236-72-7	C_6_H_11_Cl_4_O_4_P	4.03	316.90763
Di-n-butyl phosphate (DnBP)	107-66-4	C_8_H_19_O_4_P	4.00	209.09482
Diphenyl phosphate (DPhP)	838-85-7	C_12_H_11_O_4_P	3.94	249.03222
Bis(2-ethylhexyl) phosphate (DEHP)	298-07-7	C_16_H_35_O_4_P	4.53	321.22002

### 1.5 质量控制及质量保证

Di-OPEs在环境介质中普遍存在,因此为确保没有外来污染物的干扰,在样品采集、储存及提取等过程中避免使用塑料制品。所有的玻璃仪器使用前均用甲醇洗液清洗3遍,然后超声清洗,烘箱烘干,使用前,用甲醇、正己烷、二氯甲烷淋洗。

分析样本中Di-OPEs时,每批样本中包含一个空白土壤样品。空白土壤样品的制备如下:实际采集的土壤样品,经清理去除石头和残根,经真空冷冻干燥机低温干燥后,在马弗炉中进行800 ℃, 6~7 h的老化,待老化的土壤冷却至室温,加去离子水进行去活化处理后备用。空白土壤样品中检测到痕量的BCEP和DPhP,含量分别为0~0.08 ng/g和0.05~0.09 ng/g,分别占实际土壤样品含量的0~3.1%和0.7%~3.4%,远小于实际样品中的含量。在本研究中,实际土壤样品中BCEP和DPhP的含量为扣除空白背景干扰后的数值。

## 2 结果与讨论

### 2.1 固相萃取条件的优化

固相萃取基于萃取剂对目标化合物与干扰物的吸附强弱不同,对目标化合物进行分离与净化。本研究选取Oasis WAX、Oasis HLB、HP-HLB和HP-WAX 4种小柱对目标物进行分离净化。如图1所示,对于HP-WAX和HP-HLB固相萃取柱,目标物的回收率低。Oasis HLB柱对BCEP的回收率较低,回收率仅为37.51%,这可能与BCEP的水溶性较大有关。Oasis WAX柱对5种Di-OPEs的回收率为74.75%~132.96%,富集净化效果较好,因此选择Oasis WAX柱为固相萃取柱。

**图1:**
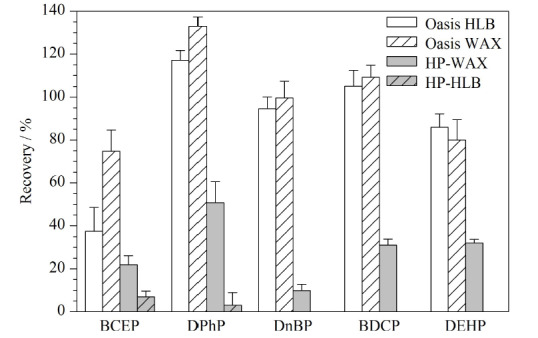
5种Di-OPEs在不同萃取柱中的回收率(*n*=3)

### 2.2 流动相的优化

色谱分析过程依赖样品与流动相和固定相的吸附-解吸差异,从而达到分离物质的目的。流动相根据化合物极性的不同,将化合物从色谱柱中洗脱出来,影响目标物的出峰时间及色谱图峰形。本研究比较甲醇与0.1、0.2、1.0、5.0、10.0 mmol/L乙酸铵水溶液5种不同流动相配比条件下对Di-OPEs的影响。当流动相为甲醇-0.2 mmol/L乙酸铵水溶液时,5种Di-OPEs的响应较高(见图2)。因此,本研究最终采用甲醇-0.2 mmol/L乙酸铵水溶液作为流动相。

**图2:**
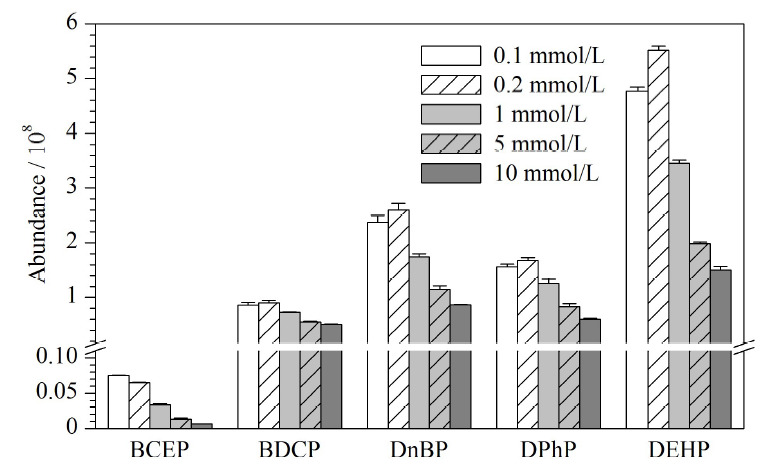
5种Di-OPEs在不同流动相中的响应(*n*=3)

### 2.3 流动相流速的优化

流动相流速对化合物的出峰时间及半峰宽产生影响,当流动相流速增大时,色谱柱压力增大,会使得色谱柱的半峰宽变窄,并且保留时间提前。本研究比较了0.15、0.20、0.25、0.30 mL/min 4种流速条件对5种Di-OPEs的影响。结果表明,随着流动相流速的增大,5种Di-OPEs的出峰时间提前,BCEP的出峰时间从1.42 min提前至0.71 min, BEHP的出峰时间从4.50 min提前至3.46 min。当流动相流速为0.15 mL/min时,BCEP、BDCP、DnBP、DPhP和DEHP的响应分别为7.31×10^6^、1.03×10^8^、3.25×10^8^、2.24×10^8^和7.79×10^8^。当流动相流速为0.30 mL/min时,BCEP、BDCP、DnBP、DPhP和DEHP的响应分别为4.84×10^6^、5.13×10^7^、1.18×10^8^、7.44×10^7^和2.83×10^8^。流动相流速为0.15 mL/min时,5种Di-OPEs的响应最高(如图3),但BDCP、DnBP、DPhP 3种化合物出现了峰展宽的现象(如图4)。因此最终选择0.20 mL/min流速进行分析。

**图3:**
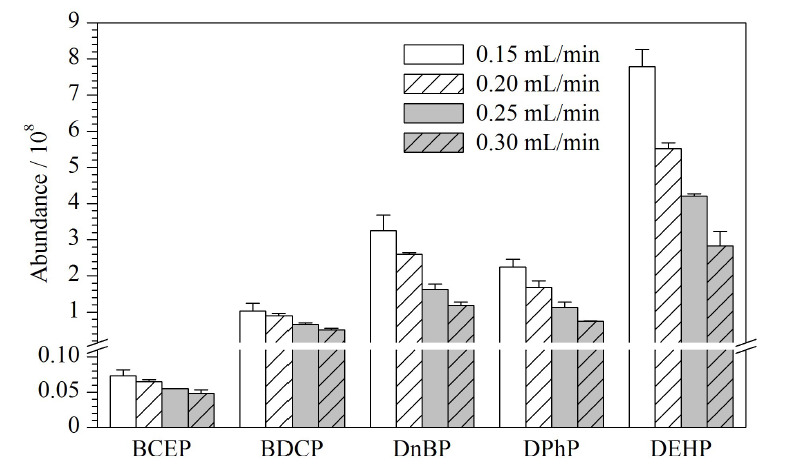
5种Di-OPEs在不同流速中的响应(*n*=3)

**图4:**
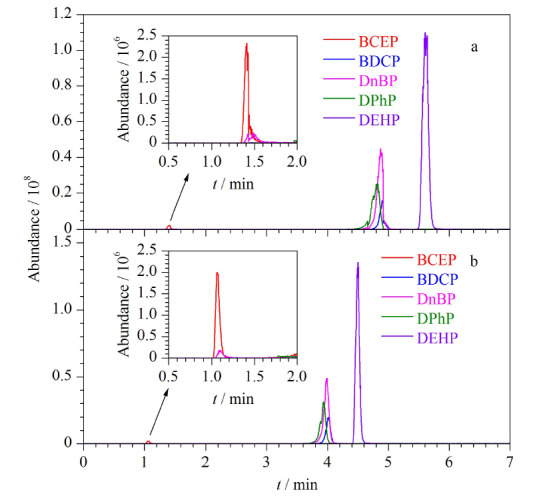
不同流速下5种Di-OPEs的总离子流色谱图

### 2.4 色谱柱柱温的优化

化合物在色谱柱上的行为是吸附-解吸的动态分配平衡过程。温度可以影响化合物在色谱柱中的吸附-解吸过程,进而影响色谱分析过程。本研究比较了25、30、35、40 ℃ 4种柱温条件下目标物的响应变化。结果表明,随色谱柱温度的升高,目标物的出峰时间提前,但总体相差不大。柱温30 ℃时化合物的响应较高(如图5),综合考虑选择30 ℃柱温进行分析。

**图5:**
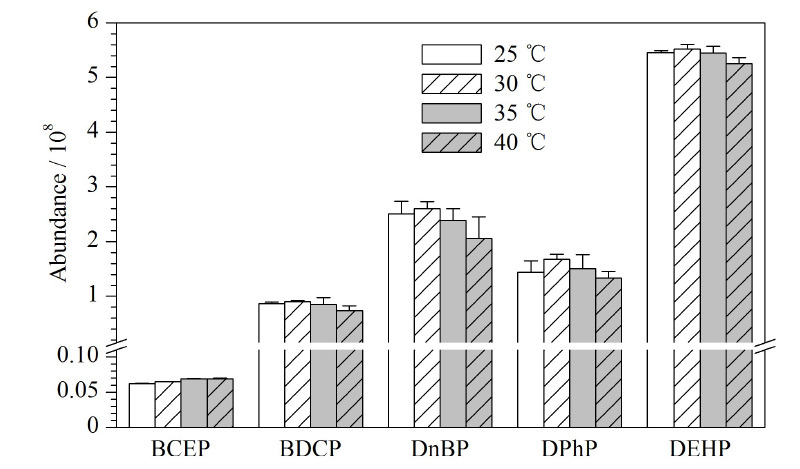
不同柱温下5种Di-OPEs的响应(*n*=3)

### 2.5 质谱条件的优化

在离子源中,鞘气有助于样品液滴挥发,帮助样品电离产生离子;辅助气在溶液去溶剂化过程中对鞘气起辅助作用,聚合样品气流,增大样品分析过程的灵敏度,因此可通过调节鞘气和辅助气的流速,优化离子束的密度和稳定性。本研究以甲醇-0.2 mmol/L乙酸铵水溶液作为流动相,色谱柱柱温30 ℃、流动相流速0.20 mL/min条件下,改变鞘气流速或辅助气流速,观察5种目标物的响应变化。辅助气流速为17.40 L/min保持不变,设置鞘气流速分别为7.51、8.58、9.65 L/min,考察鞘气流速对Di-OPEs的影响。结果如图6所示,当鞘气流速为8.58 L/min时,5种化合物的响应最高,BCEP、BDCP、DnBP、DPhP和DEHP的响应分别为6.48×10^6^、9.03×10^7^、2.60×10^8^、1.68×10^8^和5.52×10^8^。鞘气流速为8.58 L/min保持不变,设置辅助气流速分别为8.70、17.40、26.10 L/min,考察辅助气对Di-OPEs的影响。结果如图7所示,当辅助气流速为17.40 L/min时,DnBP、DPhP和DEHP的响应最高。而BCEP和BDCP的响应与流速为8.70 L/min相比,略有降低。综合考虑,本实验选择鞘气流速为8.58 L/min,辅助气流速为17.40 L/min。

**图6:**
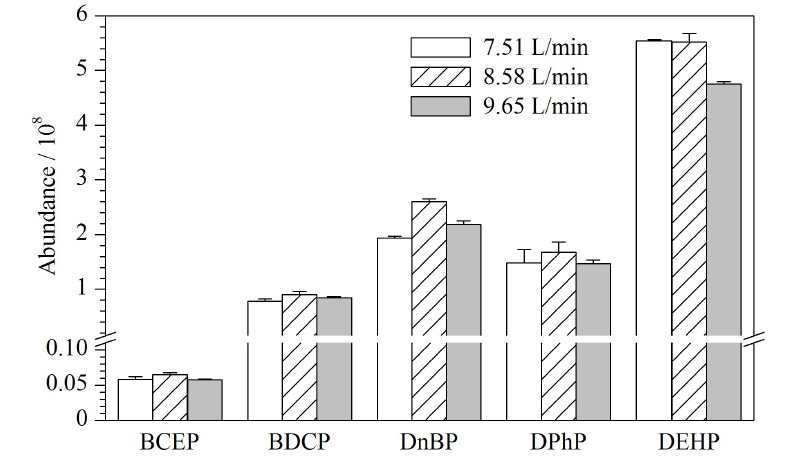
不同鞘气流速下5种Di-OPEs的响应(*n*=3)

**图7:**
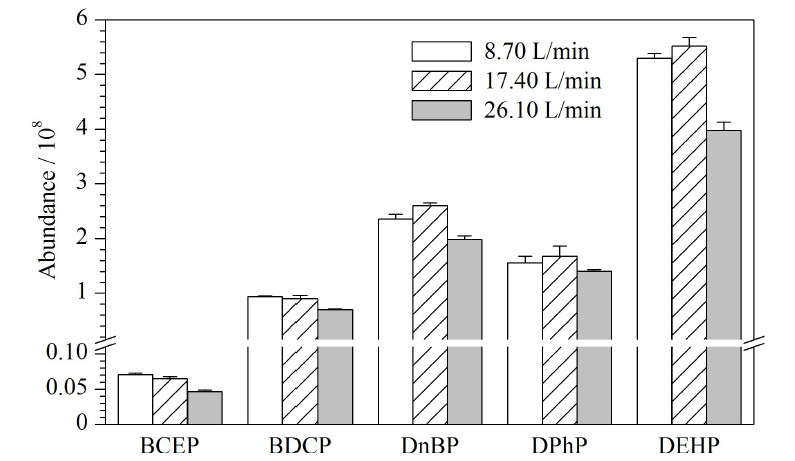
不同辅助气流速下5种Di-OPEs的响应(*n*=3)

### 2.6 方法评价

#### 2.6.1 线性范围与检出限

经UHPLC-Orbitrap HRMS测定混合标准溶液后,以Di-OPEs的质量浓度为横坐标,峰面积为纵坐标绘制标准曲线。结果表明5种Di-OPEs在0.5~100 ng/mL范围内有良好的线性关系,相关系数(*r*)为0.9985~0.9999。当进样量为10 μL时,仪器的检出限(3倍信噪比)和定量限(10倍信噪比)分别0.001~0.047 ng/g和0.004~0.156 ng/g, 5种Di-OPEs的线性方程、相关系数、检出限和定量限见表2。

**表2:** Di-OPEs的线性方程、相关系数、检出限和定量限

Compound	Linear equation	r	LOD/ (ng/g)	LOQ/ (ng/g)
BCEP	Y=1.05×10^5^X-6.41×10^5^	0.9996	0.047	0.156
BDCP	Y=1.13×10^6^X-6.04×10^5^	0.9996	0.039	0.131
DnBP	Y=2.59×10^6^X-1.11×10^6^	0.9994	0.012	0.041
DPhP	Y=1.98×10^6^X-6.78×10^5^	0.9985	0.015	0.050
DEHP	Y=4.98×10^6^X+2.04×10^6^	0.9999	0.001	0.004

*Y*: peak area; *X*: mass concentration, ng/mL.

#### 2.6.2 回收率与基质效应

准确称取2.0 g土壤样品,添加5种Di-OPEs标准品,使得加标水平分别为5.0、25.0、50.0 ng/kg,每个水平设3组平行,结果见表3, 5种Di-OPEs的回收率为56.9%~133.0%,相对标准偏差为4.4%~18.9%。该方法准确性良好,精密度较高,能够满足检测要求。

**表3:** 空白土壤样品中5种Di-OPEs的加标回收率和基质效应(*n*=3)

Compound	Recoveries (RSDs)/%			Matrix effects/%
	5.0 ng/g	25.0 ng/g	50.0 ng/g	
BCEP	56.9 (16.7)	62.5 (12.2)	74.7 (9.9)	58.3-70.6
BDCP	119.0 (4.6)	95.5 (9.5)	109.2 (5.7)	87.0-105.6
DnBP	89.7 (9.3)	83.9 (18.9)	99.6 (7.8)	71.5-86.0
DPhP	130.8 (8.4)	115.6 (13.9)	133.0 (4.4)	100.7-121.8
DEHP	74.1 (12.8)	72.0 (13.4)	80.0 (17.1)	63.0-71.7

在5.0、25.0、50.0 ng/g的添加水平下,比较基质提取液和纯溶剂中5种Di-OPEs的响应值,以评估基质效应(ME),具体计算方法见公式(1)。评价方法为相对响应值法。


(1) $\mathrm{ME}=\frac{B_{n}-B_{0}}{A} \times 100 \%$

其中,*A*:纯溶剂中5种Di-OPEs的响应值,*B_n_*:样品基质中添加相同含量的5种Di-OPEs的响应值,*B*_0_:空白土壤样品中BCEP和DPhP的响应值(在空白样品中BCEP和DPhP有痕量检出,其他化合物未检出)。

ME<100%为基质抑制作用,ME>100%为基质增强作用。ME<50%或>150%为强基质作用,50%≤ME≤80%或120%≤ME≤150%为中等基质作用,80%<ME<120%为弱基质作用^[33]^。结果见表3, 5种Di-OPEs中BDCP、DnBP和DPhP表现为弱基质效应,BDEP和DEHP表现为中等基质作用。不同地区的土壤性质存在差异,针对不同性质的土壤样品匹配相应基质的标准曲线工作量巨大,不能满足简单快速分析的要求。因此,在本研究中使用的溶剂标准曲线开展定量分析。

### 2.7 实际样品测定

本研究采集了位于河北省沧州市青县现代瓜菜示范区16个大型设施蔬菜塑料拱棚中0~10 cm的表层土壤样本,应用建立的UHPLC-Orbitrap HRMS分析方法,开展了设施菜地土壤中Di-OPEs的研究。如表4所示,5种Di-OPEs的总含量为2.53~6.94 ng/g,且5种Di-OPEs的检出率均高于60%,表明Di-OPEs在设施菜地土壤中普遍存在。其中,DnBP是设施菜地土壤中的主要污染物,含量范围为1.37~3.20 ng/g,占Di-OPEs总含量的23.4%~68.8%,中位数含量为1.75 ng/g。其次为DPhP,含量为0.47~2.44 ng/g,占Di-OPEs总含量的16.3%~35.9%,中位数含量为0.91 ng/g。

**表4:** 设施菜地土壤中5种Di-OPEs的检测结果

No.	BCEP	DPhP	DnBP	BDCP	DEHP	Σ_5_Di-OPEs
1	0.17	1.04	2.31	ND	1.28	4.81
2	0.08	0.56	2.37	0.06	0.37	3.45
3	0.09	0.53	1.85	0.06	0.39	2.91
4	0.17	1.04	1.68	0.07	0.43	3.39
5	0.09	0.58	1.37	ND	0.49	2.53
6	0.18	1.13	1.63	ND	4.00	6.94
7	0.15	0.47	1.67	0.06	0.28	2.63
8	0.30	0.88	2.03	ND	0.27	3.48
9	0.28	0.80	1.82	0.06	0.76	3.71
10	0.18	0.55	1.62	0.06	0.44	2.77
11	ND	0.72	1.67	0.06	0.26	2.72
12	0.09	1.19	1.83	ND	0.20	3.30
13	0.26	0.94	1.41	ND	0.43	3.04
14	0.72	2.44	3.20	0.06	0.44	6.86
15	0.25	1.63	1.62	0.10	1.41	5.01
16	0.57	2.19	2.66	0.08	0.79	6.28

ND: not detected.

## 3 结论

本文建立了超高效液相色谱-静电场轨道离子阱高分辨质谱测定设施菜地土壤中Di-OPEs的分析方法。该方法操作简便,灵敏度高,重复性好,一次处理即可完成多种目标化合物的分析检测,满足设施菜地土壤中Di-OPEs的测定。
